# Cancer and Chaos and the Complex Network Model of a Multicellular Organism

**DOI:** 10.3390/biology11091317

**Published:** 2022-09-05

**Authors:** Andrzej Gecow, Laszlo Barna Iantovics, Mesut Tez

**Affiliations:** 1 Independent Researcher, 999038 Warsaw, Poland; 2 Electrical Engineering and Information Technology, Engineering and Information Technology, George Emil Palade University of Medicine, Pharmacy, Science and Technology of Targu Mures, 540139 Târgu Mureș, Romania; 3 Ankara Numune Training and Research Hospital, 06100 Ankara, Turkey

**Keywords:** multicellular organism, biology, chaos, genome chaos, deterministic chaos, half-chaos, cancer, complex network, Kauffman network

## Abstract

**Simple Summary:**

Currently, knowledge on chaos has developed rapidly, and the link between cancer and “genomic chaos” seems obvious. Hopes for a deeper understanding of cancer, allowing cancer modeling, therefore relate to the meaning of the term “chaos”. It has many meanings, however. Chaos theory and medicine are conceptually quite distant, requiring the comparison and agreement of terms. This article was written by three authors whose fields cover both medical problems and complex dynamic networks suitable for modeling cancer, including chaotic phenomena. The article provides, first of all, a coherent, common interpretative basis linking chaos with modeling tools, which should significantly facilitate teams of specialists from various fields to undertake specific work on simulating cancer-related phenomena.

**Abstract:**

In the search of theoretical models describing cancer, one of promising directions is chaos. It is connected to ideas of “genome chaos” and “life on the edge of chaos”, but they profoundly differ in the meaning of the term “chaos”. To build any coherent models, notions used by both ideas should be firstly brought closer. The hypothesis “life on the edge of chaos” using deterministic chaos has been radically deepened developed in recent years by the discovery of half-chaos. This new view requires a deeper interpretation within the range of the cell and the organism. It has impacts on understanding “chaos” in the term “genome chaos”. This study intends to present such an interpretation on the basis of which such searches will be easier and closer to intuition. We interpret genome chaos as deterministic chaos in a large module of half-chaotic network modeling the cell. We observed such chaotic modules in simulations of evolution controlled by weaker variant of natural selection. We also discuss differences between free and somatic cells in modeling their disturbance using half-chaotic networks.

## 1. Introduction

### 1.1. The Aims

For the **search of models describing cancer** and its formation in a theoretical and abstract manner, expressed, e.g., by Tez in his project (https://www.researchgate.net/project/Testing-New-Cancer-Theory-Chaotic-Adaptation-TheoryCAT (accessed on 6 April 2022)) (see also [1]), one of promising directions suggested that there is **chaos**, which is generally connected to widely known ideas of “**genome chaos**” and “**life on the edge of chaos**”. These two ideas deeply differ in the meaning of the term chaos, which causes misunderstanding. The science of chaos, particularly notions used by it, is far from the scientific field of cancer, and in order to build models, those notions should first be brought closer.

For the last few years, the knowledge on chaos–life connections represented by the hypothesis “**life on the edge of chaos**” using **deterministic chaos** has been radically deepened by the discovery of **half-chaos**. It requires a deeper interpretation within the range of the cell and the organism. In this range, it has impacts on understanding “chaos” in the case represented by genome chaos. This study intends to present the **interpretation** mentioned above on the basis of which searches will be easier and closer to intuition. Half-chaos introduced and demonstrated in [2,3] significantly changes and expands the range of parameters acceptable in modeling living organisms in comparison to the preceding RBN model (RBN (Random Boolean Network) is constructed as classic “random” (er) [4] (see Section 4.1). “Boolean” means two variants of signals values, but they may not be equally probable. Such Kauffman networks were intensively investigated up until 1999, when Barabási, Alber and Jeong [5] introduced the scale-free (sf) network type) introduced mainly by Kauffman [6,7,8,9]. Many of the studies and received conclusions based on the RBN, including mainly the GRN model (GRN is an RBN network interpreted as a genome. Each gene can be in one of two states: active or inactive (see Section 1.4)) and its consequences, require taking new circumstances resulting from half-chaos into account.

The RBN model led, more than 3 decades ago [8], to the well-known Kauffman hypothesis (life on the edge of chaos and order), but it was a model that was strongly simplified in important aspects. The analysis of the basis of these simplifications led to a significantly in-depth model, the conclusion of which was, e.g., the reformulation of the above-mentioned hypothesis into the following: **life evolves in half-chaos of not fully random networks**. Half-chaos turns out to be the basis of system stability and exhibits a surprising number of basic features of living organisms, and these do not need to be additionally assumed, which makes this state of the system a promising tool for modeling.

**Genome chaos** (also known as chromosomal or karyotypic chaos) is more close to the carcinogenesis problem [10,11,12]. Current differences between deterministic chaos used in the mathematical theory of chaos and chaos used in “genome chaos” may be decreased by using observations in the simulation of half-chaotic, growing, open systems described in [3], where large modules falling into chaos compete with half-chaos.

Shapiro [13] presented an interesting study on the chaotic nature of genome chaos. The study shows that cancer genomes can experience significant restructurings that can evoke the most significant stages of tumor development in many chromosomal locations. DNA restructuring and the way in which the chaotic restructuring of the cancer genome behaves have been studied in terms of cellular and molecular processes. The conclusions indicate that genome restructuring may be appropriately reproducible to allow potential therapies that stop tumor development.

Multiple myeloma disease is described by severe chaos in genomic abnormality. Li et al. [14] outlined that multiple myeloma is characterized by genomic chaos. This fact makes it hard to differentiate passengers from drivers in mutations. Genetic abnormalities in patients with multiple myeloma have been shown to display 80 unique protein network signatures. This aspect can be utilized for applications in current therapies targeting key pathways and in the advancement of innovative therapeutics.

Russo et al. [15] studied **epigenome chaos**. In that study, 83 epigenomic and genomic events that arise in cancer were analyzed. New methods of studying the epigenetic difficulties in a population of neoplastic cells were also discussed.

### 1.2. Structure of This Article

This article is interdisciplinary and is dedicated especially to cancer researchers who are trying to describe carcinogenesis in terms of complex network and chaos. However, such aims need a group of specialists from different disciplines, and the participants should use the same language. The notions used in these two disciplines are different, which can cause misunderstandings and requires a common basis. This article is written by such a group of specialists from different parts of the world in order to provide assistance in understanding each other. Therefore, for some readers, only some sections of the article will be interesting, and other readers, other sections of the article will be interesting. In particular, Section 4 is more technical and is intended for specialists in chaos, complex networks and simulations; it is not needed by medics. Figures are technical elements, but they are also included at a simplified level. They belong to chapters, where their main aspects are descripted in detail. Notions and terms are used on an intuitive level in the first few sections in common text (Section 1, Section 2 and Section 3); while this is enough for medical personnel, it is not enough for complex network specialists.

In Section 1, only basic initial circumstances are clarified. Typical sequential description is used, inter alia, in the science of complex networks. It is: First, the description of the theory (here, the half-chaos) and, then its interpretation. We understand that such a typical sequential description would not gain enough credit for the patience of medics. Therefore, the description of half-chaos is successively introduced together with an interpretation, its increasingly visible needs and the complexity of model. This is introduced in Section 2 and Section 3. We hope that, in this way, medics will read the article up until the end of Section 3 and that they may even read some elements discussed in Section 4. A summary in Section 5 is also provided to all readers.

For complex network specialists, since they are modeling cancer or other biological problems, not many details on cancer and other biological information are incorporated into Section 2 and Section 3.

### 1.3. Meanings of the Word “Chaos”

There are many very different meanings of the word “chaos”. **We deal with well-defined deterministic chaos. Its basic feature is possessing great sensitivity to initial conditions.** Of course, this formulation contains many assumptions that need to be explicated, but these are usually not a problem. The meaning of the term “chaos” in this article will be discussed in more detail below in Section 1.4 as “deterministic chaos”.

‘Chaos’ as a word that usually means a lack of a visible rule for a complex phenomenon. Disorder, confusion and anarchy are given as synonyms. Until we find a rule, we assume that it is a random phenomenon. In this sense, the term “chaos” enters science. It is used in descriptions of various phenomena and their circumstances. To indicate the meaning of a given statement, an additional term is usually added, e.g., “genome chaos”, “chromosomal chaos”, “karyotype chaos” or, in another role, “chaos game” (method).

“The term “**chaos game”** originally referred to a method of creating a fractal, using an initial point selected at random inside it. The term has been generalized to refer to a method of generating the attractor, or the fixed point, of any iterated function system. The chaos game method plots points in random order all over the attractor. This is in contrast to other methods of drawing fractals, which test each pixel on the screen to see whether it belongs to the fractal.” [https://en.wikipedia.org/wiki/Chaos_game (accessed on 23 December 2021)]. In this case, “chaos” was used in the sense of a lack of an easily visible rule when a new point is drawn at random. There is no great sensitivity to initial conditions (the main deterministic chaos feature); moreover, there is an opposite feature in which (semi-always) the same attractor undependably results from the first point in such an iteration.

“**Genome chaos** (also known as **chromosomal or karyotypic chaos**) is observed both in experimental systems and clinical samples, particularly in cancers with elevated genomic instability (e.g [16,17]). Here the term genome chaos is used to refer to the process whereby highly altered, chaotic genomes are formed and the dynamics of continuous change that leads to their formation. Chaotic genomes can be defined by both extreme structural and numerical alteration.” [10]. More simple definitions may be found on the Internet: “The terms ‘**genome chaos**’, or ‘**karyotype chaos**’, have been coined to describe fast and massive genome or chromosome re-organization under crisis.” [https://www.frontiersin.org/research-topics/12938/the-interplay-between-chromothripsis-genome-chaos-and-cellular-crisis (accessed on 23 December 2021)]. Here, the meaning of “chaos” is closer to the lack of known rules than to “deterministic chaos”. There is a possibility that those rules are deterministic (for now they are under investigation), but the main deterministic chaos feature is questionable here. Typically, an entire mechanism (from the circumstances of its initiation to its results and role in genome adaptive evolution) is additionally included in the term “genome chaos”. Genome chaos is an old mechanism used by bacteria [18,19] and eukaryotes as the last chance to survive. “Genomes chaotic reorganization is crucial for the surviving of the system. Phenotypic diversity created by genome chaos can be advantageous in fluctuating environments and is well established in the ecology and population genetics literature [20]. It has been suggested that chaos can act as a «heterogeneity engine» that allows a population of cells to quickly explore a large number of phenotypes (different morphology, nuclear structure, chromatin architecture, metabolism, trans-membrane potentials etc.)” [1,21]. Genome chaos contains a large random (may be not fully random) change; then, many descendants are produced, and the great majority will die but perhaps some will survive and adapt to the new environment. Therefore, this mechanism is purposeful, which is not the general feature of chaos.

A discussion including a new proposal for understanding the term chaos in the field of “genome chaos” will only be possible after reading a more detailed description of deterministic chaos and half-chaos (see Section 2.6).

### 1.4. Deterministic Chaos

The term “**deterministic chaos**” means that unambiguous iterative processes starting from two very close initial points produce maximally different results. For functions defined in a infinite and continuous space in mathematics, there is a **chaos theory that developed based on Lyapunov exponents**. Mathematicians consider deterministic chaos typically only in such a space. However, most interesting systems, such as living entities, human-made artifacts or institutions, are large, complex, discrete and finite dynamical networks. There are no mathematical tools for such a space and mathematical investigations are based here on approximation, allowing the use of methods developed in chaos theory. Such an approximation, however, is a dangerous simplification [2,22,23] that can miss important phenomena. The alternative method is a computer simulation, which is widely used.

In the discussion above, the term “complex” connected to the term “chaos” already appeared as an important feature for the second time. “Complexity characterizes the behavior of a system or model whose components interact in multiple ways and follow local rules, leading to nonlinearity, randomness, collective dynamics, hierarchy, and emergence.” [https://en.wikipedia.org/wiki/Complexity (accessed on 25 May 2022)]. There are many approaches to characterizing complexity in science; in [24], many of these can be found, as well as in special issues or a book edited by Iantovics et al. [25,26,27]. In [28,29,30] and especially in [31], a complexity threshold was shown. In biological and artificial computational systems, structural and functional complexities can enter intelligence. In [32], a complex, artificial, agent-based, medical diagnosis system was proposed. In [33], a metric that is able to measure the intelligence of artificial complex systems is provided.

Computing capabilities of computers have grown rapidly over the past few decades, allowing the simulation of more complex models. In 1990, Kauffman [8], based on simulations and the support of chaos theory, proposed his famous hypothesis: “Life exists on the edge of chaos and order”. **His model, using finite Boolean networks, was based on the assumption that a network is fully random**, which allowed for easier simulations and theoretical studies of complex networks. **Such networks may be only either “ordered” or “chaotic”**, with quick (narrow in a watched parameter) phase transition between them. The area of parameters space near the point of phase transition between order and chaos is called the “edge of chaos”. Parameters of such fully random, chaotic networks are called “**chaotic parameters**”.

In [22], a critique of three basic assumptions used by Kauffman is openly provided:

1. Networks describing living entities are not fully random, especially in the stability aspect. This aspect is connected to notions chaos and order, because the stability is an effect of natural selection, which leaves more stable entities.
2. Negative feedbacks have been incorrectly taken into account in the statistical study of their effects on post-disturbance stability, but they are commonly considered the basis of the homeostasis of living objects. Their proportion should be much greater than random [34,35]. In Kauffman’s model, this surplus over randomness does not have the most important property: it cannot go beyond the range of correct operations, but this is the main reason for the loss of stability after a random disturbance.
3. Using two-valued signals, as is in Boolean networks, is too extreme of a simplification that has a significant impact on statistical conclusions, and we should consider signals beyond two-valued signals.

This critique is supported by estimation from observation [2,22] of really random disturbances in nature; therefore, networks describing living organisms should have parameters as fully random chaotic networks, which entails being far from the edge of chaos. The most discussed parameter, “connectivity” (in Section 4.1 introduced parameter K), is typically estimated from nature and is greater than what Kauffman’s model [36,37] allows. Demonstrated from experiments with living organisms, the life normally modeled by GRN (Gene Regulatory Network based on RBN) is on the edge of chaos or in an ordered phase [38,39,40,41,42,43], and this is based on the above-mentioned incorrect assumptions; first of all, it does not correctly take into account regulatory mechanisms.

Based on such remarks, a large series of simulation investigations was conducted and published in [2]. **Finding of half-chaos** is the main result of these investigation of source of significantly larger stability of some part of systems with “chaotic parameters”. This leads to a deep reconstruction of Kauffman’s hypothesis: “**life evolve in half-chaos of not fully random networks**’. Half-chaos (probably) cannot be seen in typical chaos theory, because it is based on the features that are “discrete” and “finite” relative to a network. Short attractor measured in discrete time steps in network calculations is its basis. **Being limited to small attractors is the only deviation from randomness that defines half-chaos**. In such a short attractor, the probability of a secondary meeting of an initially disturbed discrete state is significantly smaller than in a typical long attractor; this introduces a chance for damage to fade or stay small.

### 1.5. Main Feature of Half-Chaos

Features of half-chaos are particularly interesting when describing the evolution of living organisms. First of all, it offers the Darwinian mechanism not as an additional available assumption but as its main internal consequence of the short attractor assumption. It needs only an addition of reproduction as a result of a system’s function. In the emergence of Darwinian mechanisms, we encounter the natural criterion of identity, the natural criterion (threshold) of small changes in functioning (small damage) and, in effect, also a model of death (elimination). We can say that life is the maintenance of half-chaos while a system is exposed to random changes [2,44]. It is based on the evolutionary stability of half-chaos: If disturbance initialize small changes in functioning (small damage), then the system stays half-chaotic [2], and such disturbances may stay in the system as evolutionary changes. After a small change of functioning, the system functions similarly, and therefore it remains itself (identity criterion). Damage as an effect of random disturbance may be either very small (ordered change) or very large (chaotic change near Derrida chaotic equilibrium [45,46]) in similar share. If the damage is chaotic, then the system works completely (maximally) differently than before; the system ceases to be the same system. If one change (disturbance) in the system produces large damage and remains in the system, then it falls into normal chaos practically without a chance to return to the similar way it functioned before; this means that it loses half-chaos. This is a model of death and elimination.

Half-chaos may be experimentally detected by measuring the distribution of the damage size after random small disturbances (initiation of damage). Such distributions (see Section 4.3) contain two peaks: the left one comprises very small damage (ordered) and the right one comprises very large damage (chaotic). Between these peaks, there is a large gap; there are no such events in this gap. This gap ends the left peak comprising small damage in a natural (but statistical) way and makes this way a threshold of small change (of functioning, i.e., damage) and a criterion of identity. For fully random particular network, there is only one peak—either the left peak for an ordered network or a right peak for a chaotic network. Each half-chaotic network has both these peaks with a similar volume.

A more detailed but still simplified description of models, networks and experiments of half-chaos investigations is provided below in Section 4. Full existing descriptions contain documentation [47,48] and articles: longer manuscripts [2,3] and shorter article [23,49].

## 2. Free Cell without Meiosis as a Half-Chaotic System

### 2.1. Basic Simplifying Assumptions

The half-chaotic system is a good model of a living organism, but both of these terms refer to very complex entities, and in order to connect them, extremely simple cases should be initially taken into account. In a study introducing half-chaos (Gecow 2021), an autonomous system was investigated, i.e., without connections with the environment. It is clear that a living organism is highly dependent on the environment—both its current metabolism and its evolution. The assumption of autonomy is the assumption that the environment is so constant that its influence can be neglected. The study that takes into account the influence of the environment on half-chaos was initiated by the publication [3], but it only studied the ability of a system to maintain the half-chaos based on the assessment of the size of the change at the system’s outputs. This is only an introduction to the study of adaptive evolution, still with established signals from the environment. These studies turned out to be very complex, and many observed phenomena required deeper investigations. One of the most important effect is the effect of spontaneous modularity (growth of large modules) during the growth of the network describing the system that requires more complex models. However, this effect can already be implemented in a deeper understanding of genome chaos (see Section 2.6).

Research on the adaptive evolution of a complex network was previously carried out with a highly simplified algorithm [50,51,52,53,54,55,56], and it showed the presence of many “structural tendencies” corresponding, inter alia, to known classical regularities of the evolution of ontogenesis (e.g., terminal addition, terminal changeability). Among these tendencies is “covering”, which results from environmental variability, i.e., significant changes at the inputs of the tested system. The results of the covering study have not been widely published thus far, and they are only mentioned in [50,54,55,56,57], but recently, a broader description of these studies has been published temporarily in the form of a preprint [44]. The obtained conclusions are convincing, and they indicate regularities similar to cenogenesis [58], which has so far been poorly indicated in the literature with repsect to the regularities of ontogenesis evolution. Research using more advanced algorithms, which have been enabled by the rapid development of computer capabilities, has already resulted in the detection of half-chaos and should further deepen the knowledge of covering as a specific form of evolutionary adaptive changes.

The assumptions of the features of the modeled organism should also be extremely simplified. In this chapter, we will limit ourselves to an organism—a single cell, e.g., bacteria—without the exchange of hereditary information between independent cells reproduced vegetatively (e.g., by dividing). This assumption not only excludes meiosis with fertilization but also the exchange of genetic information through plasmids. This greatly simplifies theoretical considerations, but it seems that little important factors are lost in terms of the aspects considered.

An organism defined in this manner does not experience “death from old age”; its life cycle is a closed loop, and the fitness assessment covers the entire life cycle [56]), which can be identified as the attractor when a network models this organism. It will not be so simple when we turn to a model of a multicellular animal organism, in which its trajectory (not the infinite cycle anymore for soma cells) is not closed for the soma, and a large part of this trajectory is not under selection control. Before we move on to discussing the model of such an organism (only there can we talk about cancer), we need to be clear about how the above-defined simple cell is modeled, i.e., how the interpretation of the elements and phenomena of a half-chaotic system, mainly an autonomous one, looks like in this case.

### 2.2. Evolutionary Stability of the Half-Chaos

Let us recall the basic features of the half-chaotic system, which we will shortly compare to the corresponding features of the modeled cell. A small disturbance of the system causes a change in its functioning. Both of these changes should be distinguished. The nature of this disturbance can be any, and we focus on a slight permanent change in the structure of the system. The resultant change in functioning called “damage” in a half-chaotic system can either be very small or very large; medium damage is practically non-existent. Both of these possibilities occur with a similar probability, but this conclusion applies to a system **without regulatory mechanisms** supporting half-chaos. Such mechanisms are mainly based on **negative feedback**. (Section 4.3 and Section 2.3).

A typical course of great damage is a sudden avalanche that increases in a very short period of time to a maximum level determined by the level of Derrida’s chaotic equilibrium. We call it an “explosion into chaos” (see Section 4.2, especially “crocodile” b there). Such an explosion does not have to occur immediately after the disturbance, and sometimes the damage stays low for a while and then suddenly an explosion occurs; however, the longer the period of time for low levels, the less often it occurs. Most will explode almost instantly.

A chaotic system in the normal sense of deterministic chaos is the effect of such an explosion. It functions very differently from the system before the disturbance. Despite the similar or even the same structure, it is a completely different system—it loses its identity. Such a large change in function corresponds to the death of the modeled cell (Darwinian elimination); all mechanisms of supporting its life worked out by selection function quite differently and result in completely different effects.

Small damage (also called “**small change**”) does not proceed from half-chaos to chaos; that is, there is no chaotic explosion here. This is a feature called, in [23], “**evolutionary stability of the half-chaos**”. The system, although slightly changed, remains very similar, functions very similarly, and remains itself. A large gap between a small and a large change in the distribution of the size of the change objectively determines the “small change” and “natural criterion of the identity of an evolving object” in a natural way.

The primary reason for protection from explosions into chaos is the short attractor that defines half-chaos. Damage can either expire or remain low for some time, as in the above-discussed delayed explosions. The change in structure (this disturbance) causing damage, however, still exists (“permanently”) and is sometimes a source of a **secondary initiation** of damage in circumstances different than before. After the rotation of the attractor, no other circumstances occur, and if the initiation is repeated, as if stumbling over a permanent disruptive change, the damage will expire in the same manner as it did previously in first attractor rotation.

In addition to the first attractor rotation, there is also a path to this attractor that extends the dangerous time until the end of the first attractor cycle. The attractor is usually changed due to the construction change, and the new state of the new system is not immediately present on the attractor.

### 2.3. Regulation

A very important factor is the **regulation** commonly present in living organisms, which is called homeostasis. Its main form includes **negative feedbacks**, the auxiliary influence of which was investigated and described briefly in Section 4.3 and more precisely in [16,23]. Regulation can radically reduce the likelihood of an explosion into chaos (as shown in Section 4.3), and it is widely recognized that it is very intense in living organisms. There are even definitions of life based on negative feedbacks [34,35]. Regulatory mechanisms are reactions to various deviations from the norm that eliminate these deviations. They are purposeful information (towards the goal of “continue to exist”) acquired through natural selection. It is almost the same as “biological information”. Such information indicates a construction that more often obtains the “goal”. A measure of the amount of information, also on purposeful information, is a form of entropy, but in theoretical and statistical terms, it does not require taking energy into account. A goal not based on an intention and purposeful information was defined in [59,60] to describe nature when men did not exist yet, and on this basis, the Darwinian mechanism and the definition of life were derived (in an alternative way than from half-chaos).

Regulation usually has a range within which it functions properly to cover typical fluctuations that require adjustments. However, the possibility of fluctuation above the correct response threshold remains. The selection makes sure that this range is large enough, but there are many factors that usually leave the possibility of exceeding it. In the RBN model, Kauffman took into account negative feedbacks, but without the possibility of their regulation going beyond the range, inserting them as “**ice**” (no change of the node state). Ice naturally persists in an ordered system, and Kauffman interpreted them as “order for free” [61]. The area of system parameters where ordered systems are located is, however, particularly unique and rather inconsistent with the estimates of these parameters by the nature of the modeled organisms [37]. Moreover, the idea that the accumulation of purposeful information is the result of a random process without selection, to which “order for free” is prevalent, is in conflict with the widespread increase in entropy in random processes. Order for free, therefore, concerns a different aspect of order compared to biological information (or purposeful information).

### 2.4. Source of Variation

**Exceeding the control range as the disturbance-initiating damage discussed above will not occur in a deterministic autonomous system on the attractor. A typical cause is the influence of the environment**, which has gone beyond the parameters for which the selection has prepared the organism. The disturbance that initiates damage in an autonomous system is, therefore, a simplified consideration of the environment. One should distinguish between a single, temporary exceeding instance of the control range, which is not permanent, and a permanent change in structure because it has a significant impact on the basic mechanism of half-chaos and evolution. If the duration of the environmental signal exceeding the control range is greater, comparable to the average time of the secondary damage initiation, then it can initiate more than one different form of damage. Therefore, it should be treated as a permanent change, and the short attractor is an important feature. Kauffman [9] studied single (temporary) initiations corresponding to very short fluctuation times of the environmental parameter disturbing the functioning of the system. After such a disturbance, the orderly system usually returns to the attractor from which it had precipitated, even without changing its phase. The mechanism of reactions after a temporary disturbance is important for the effectiveness of regulatory mechanisms after a permanent disturbance, which results in exceeding the regulation range as a temporary disturbance. In the real organism, damage usually encounters many regulating mechanisms that make it difficult to achieve chaos (see model “a” in Section 4.3), but when the extent of their correct operation is exceeded, the damage can increase.

A permanent change in the system creates evolution. If the new organism functions similarly and has not lost its half-chaos, the modeled cell continues to live and reproduce. If the environment has recovered, it can trigger a new disturbance. The “small change condition” is not the same as the “adaptation condition”: It is weaker, but it contains much of what we expect from the adaptation condition. It seems that real natural selection works precisely on this weaker condition, and genuinely adaptive changes that not only allow survival in new circumstances but also a return to previous ones have a better chance of staying longer, but they are less likely to occur.

**Changing environmental demands is not necessarily the cause of a change in organism composition**, and these changes may even be expected to be rare overall. Such a change in the environment permanently disturbs functioning and either the organism will survive (as a half-chaotic system) or it will not; i.e., it will fall into chaos and it will die. For evolution to take place, there must be a permanent change in the structure of the system. **In such a case, the model shows only one source for the construction of change—it must be performed by a separate act in the environment.** (In the analyzed model, we have excluded crossing-over). This, however, is the result of simplifications of the model, which at the present stage of research is based on the Kauffman network. The structure of the network is given. In the case of growing networks, the disturbance in the form of adding or removing a node is admittedly random, but it is also imposed from the outside as a simplified influence from the environment. There is currently no possibility of creating a network structure in the model as a result of the functioning of this network; such a possibility is very important, but it remains a challenge for future researchers. It should be expected here that a disturbance in the system’s functioning by environmental factors will cause changes in the resulting structure of such a self-building network; thus, its permanent change, which will cause another damage that requires assessment, and, that half-chaos is also possible in such networks. This problem emerges especially when we model the ontogenesis of multicellular animals or even the cycle of a change in structure of one cell. There are two “dimensions of time”: metabolism and structure development, which differ in time period lengths (see discussion in [44] Figure 11).

Add the possibility of a **prepared reaction** (on environmental change) to the already indicated separate act of the environment in the form of a reconstruction of certain aspects of the construction, when a change in the environment requires some construction change. In the case of a previously known stimulus, these are normal responses to an environmental signal, including regulatory responses. In evolutionary biology, these are plasticities. Their source does not all see in earlier natural selection. Jablonka called these “**Lamarckian mechanisms**” [62,63,64,65]. Now, it is referred to as “developmental bias” [66,67]. In both cases, this caused widespread discussions [68,69,70] related to the ideological foundations of the rationality of explaining these phenomena.

When an environmental problem is encountered for the first time, in order for the system to be able to adapt to new circumstances, there is also a need to induce a permanent change in structure. The change must be random; however, its range does not have to be. This and the emergence of plasticity cannot be modeled with the Kauffman network; it has to be a self-constructing network. **Such reactions include “genome chaos”**; therefore, even the title of [71] contains the “Lamarckian view”. Hengs [72] see genome chaos as an important avenue in the search for new concepts in understanding cancer.

Genome chaos is observed in free-living cells [18,19], and it can be considered an acquired feature grounded by selection [64]. The term “chaos” in the term “genome chaos” is used in a different sense than in the clear term “deterministic chaos”, and the conclusions drawn in a study of deterministic chaos cannot be simply applied. Its meaning was distinguished from deterministic chaos at the beginning of the article, but it will be soon discussed in Section 2.6.

The determinism feature of the considered network (implying form using of deterministic chaos) that models a living organism is also an approximation. Real mechanisms are statistical (stochastic) in nature, and as such, they have the right to spontaneously go beyond the range of correct operations and initiate damage. Thus, the sources of the change in the structure, which is the initiating disturbance, may be varied.

### 2.5. In-Ice-Modularity

To define half-chaos, the short attractor is sufficient; however, we consider a variant of half-chaos, which starts its evolution from a point attractor state (**PAS—Point Attractor System**; see Section 4.1). The left peak of the damage size distribution is different from when the evolution starts only with the assumption of a short attractor than when it starts from the point attractor state by forbidding attractors that are too small (including point attractors). In the first case, there are only a few changed node states in the left peak: practically only two and most often zero. In the second case, even relatively large damage occurs, and it is still far from the right peak, but the greater the damage, the less likely it occurs (it is a faster decline than a linear one). Such a picture is more similar to reality than the extremely narrow peak of the first case. Small permanent disturbances in the system (without additional regulation) with a point attractor in about 98% are also provided by a system with a slightly different point attractor; thus, in order to proceed to a more natural state with a small attractor, prohibiting extremely small attractors is necessary (only leave initiating changes when the attractor is greater than, e.g., 6, but the damage stays in the range of the left peak). A cell cannot stay in only one point attractor state continuously as reproduction occurs in its life cycle, requiring changes in the state of the network model of the cell.

There is another important feature that distinguishes both types of half-chaos—the occurrence of “**in-ice-modularity**” in the type started from PAS. The presence of in-ice- modularity defines a wider type of half-chaos than that initiated by PAS. Networks with the same properties can be built according to the recognition of a system’s features after a certain section of evolution starts from the point attractor. These features comprise “in-ice-modularity”. Therefore, such a network does not have to start from PAS.

The point attractor system (PAS) comprises a lack of change in the state of the network; i.e., the function of the network does not change the state of any node. A node that does not change its state is called **ice**—its state is frozen. The point attractor network comprises only ice—it is completely frozen. Contrary to appearances, this state of the network has a good interpretation; the network still functions in the same manner. It is a model of life-sustaining metabolism. Apparently, nothing changes, but the network functions according to normal rules, which also apply in a chaotic network in which there is practically no ice at all. Moreover, in the first case of a random network only with a forced short attractor, ice is not statistically present.

Distinguishing between metabolism and structural changes, when the function of a network is to model the life cycle, is a deeper interpretation problem. Both of these aspects differ mainly in the time scale of the node’s state changes, as if creating two dimensions of time. This problem is discussed in more detail and is presented in a figure in [44] Figure 11. Undoubtedly, the nodes carrying out metabolism participate in structural changes, but currently, the Kauffman network formula does not offer the possibility of reorganizing the structure (connections and number of nodes) through the function of this network.

Kauffman placed life on the border (edge) of an area of orderly and chaotic systems where ice is abundant. “Living” systems have either lakes of activity in ice on the ordered systems side or on the chaotic side; when they come together (percolated), they have a large amount of ice that inhibits the spread of avalanche of damage. The ordered system was practically composed of ice, but in Kauffman’s model, the ordered or chaotic state resulted from the parameters of the random system, e.g., the number of inputs to a node (called connectivity, which was usually fixed in a given network, and the number of output links was variable). The half-chaotic system obtained during evolution starting from the point attractor also presents a similar picture to systems near the order-chaos phase transition in the Kauffman model, but it has parameters such as highly chaotic systems (i.e., chaotic parameters). The half-chaotic system can become chaotic without changing these parameters, which is a model of death. The parameters of the systems estimated by the nature of the modeled organisms lie in the area of “chaotic parameters”, which was noticed by many researchers (e.g., [36,37]), but the Kauffman model did not allow for such parameters, because the properties of the chaotic system are drastically different from the modeled organisms. Kauffman’s model is based on researching living organisms [39,40,41,42,43], but he retains a model where the network is fully random.

Small lakes of activity are split by ice that actually impedes tearing damage. They function as well-separated modules, but this separation is not a result of a different density of links between them; that is, it is not a feature of the connection structure but a feature of functioning. Thus, it is a different form of modularity than the classical one; in [2], it was called **in-ice-modularity**. Such modules are statistically related to classical modules that may facilitate their creation. Each network (except for those in which all nodes are connected with all other nodes) has heterogeneity in the nature of weak classical modules. In-ice modules allow small attractors even in a large network. Usually, there are several such modules; each has a small attractor, but they are usually of different lengths. The entire network attractor is a composite of these attractors, and it can even be large, but the half-chaos maintenance mechanism works on small attractors in these in-ice modules.

### 2.6. Chaos in Term “Genome Chaos” as “Deterministic Chaos” in Module

Now, we can return to the problem of the meaning of the word “chaos” in the term “genome chaos” and compare it to described deterministic chaos from the point of view of half-chaos. Genome chaos is an important term: “genome chaos research has led to the important concept of System Information Self-Creation Under Crisis, which will have a profound impact on cancer research and evolutionary biology”. [72]

“Genomes chaotic reorganization is crucial for the surviving of the system.” This quote was already mentioned in Section 1.3 from [69]. The quote means that genome chaos helps a system’s survival, but an explosion to deterministic chaos in the view of half-chaos is a model of death (Darwinian elimination). By considering one cell falling into genome chaos, as Barbara Wright [18,19] had described, it functions similarly to previous cases in which it can reproduce—it remains as the same cell (identity criterion: it remains half-chaotic and not chaotic in the sense of deterministic chaos) but is a little bit different. Part of this cell functions differently than it had previously, but this only applies to a part of the cell, for the other parts function in the old manner. If these two parts did not influence each other (so much that deterministic chaos occurs only in one of them), then they are two semi-independent modules. These modules may be classic modules or in-ice modules.

In [3], large modules were observed that fall into chaos after disturbance, but they are smaller than thresholds of small change and, therefore, such cases of permanent initiations were accepted as evolutionary changes. After such changes, half-chaos remains, which should be interpreted as the fact that the modeled cell survives. However, in this module, chaos also remains. Often, the attractor is longer than the tested period. In such a case, the certainty that the entire system (model of one cell) would not proceed into a chaotic state for a longer period of time than the tested section was significantly lowered. The time required for an explosion into chaos may be so lengthy that it may contain a few cell reproductions (on interpretation levels). For a fully deterministic network, if new, external, different initiations do not occur, all daughter cells will have to die at once, but we know that, in reality, we are dealing with stochastic determinism and their trajectories may differ.

Such chaos in the module may be an effect of a previously unknown factor in any aspects of environmental disturbance. In the simulation, it was a random disturbance. However, some aspects of the environmental stimuli may be identified, and according to them, particular reactions can be applied. It could be some (currently unknown) mechanisms permitting, in a defined module, some random (or even semi-random) changes that previously were blocked, e.g., by repairing mechanisms. For example, in the case described by Wright [18,19], it seems that such mechanisms are prepared as a particular purposeful reaction that is able to indicate the locations in genomes that will be modified. As such, it is a Lamarckian mechanism, as discussed in [62]. Using the term “Lamarckian” is dangerous because it is typically understood in an incorrect manner, as shown in [69,70].

Problem of randomness source of allowed changes remains. They should have an internal cause; however, they have not to. Changing some controlled parameters can open the gate to chaos (in defined module). This is one possible interpretation of genome chaos; however, there still remains the possibility that there is no chaos (in view of deterministic chaos) but only random mechanisms such as crossing-over.

### 2.7. Summary of the Description of a Free Single Cell

A free living cell without an exchange of hereditary information, reproducing by division, is well modeled by an autonomous half-chaotic system. Its entire life cycle is under the control of natural selection. The natural selection here is to maintain half-chaos by a small change in functioning (evolutionary stability of half-chaos). There is a short attractor for the life cycle. Death from old age does not occur here but only as a result of a disturbance, and the source of this disturbance is the environment (in fully deterministic model); however, it is not explicitly present in the model. Changes in the structure within the life cycle are the result of functioning and constitute this visible attractor. Thus far, they have not been modeled as structure changes because researchers do not have a model in which the structure of the network can be a result of its functioning. There have been attempts in virtual machine research; for example, [73,74] and computational and hyper computational theory [75] are examples of such attempts. However, those are still in the early stages and cannot model the complexity of real biological systems. For our study, we assume that the properties of such a self-building network are similar to simpler networks that we have simulated. We have demonstrated that there is half-chaos in those simpler networks, and that the same properties will be exhibited for biological life-cycle networks. This, however, requires further research. The formal process algebra provides a promising direction and formalism [76]. Disturbances cause a change in functioning—damage. Damage can be either very small—the system survives while remaining half-chaotic—or very large—the system dies and becomes chaotic. This is the natural criterion for the identity of an evolving system and the evolutionary stability of half-chaos.

Regulatory mechanisms, i.e., reactions to deviations from the norm that eliminate these deviations, play a very important role in maintaining the stability of functioning, including half-chaos. Usually, these are negative feedbacks. Regulatory mechanisms support half-chaos resulting from the short attractor (see Section 4.3). They constitute purposeful information (corresponding to biological information) acquired through natural selection. The range of proper functioning of these mechanisms is usually limited and can be exceeded, which is an important factor that results in out-of-control damage and can turn into an avalanche—an explosion into chaos.

Reaction to environmental stimuli may be a case of regulatory mechanisms even if such environmental changes are not fully known. Such unknown (in most aspects) environmental changes are stress factors, which may lead to genome chaos. However, this chaos is not in the entire system (cell) but only in some module defined by regulatory mechanisms using known aspects of encountered environmental change. Such regulatory mechanisms prepared by natural selection that change the property and construction of a cell (system) in a purposeful manner are called Lamarckian mechanisms. In cases of genome chaos, a part of these mechanisms is purposeful, but the second part creates random changes that can lead to new adaptive properties.

## 3. Half-Chaos in Modeling a Multicellular Animal Organism

### 3.1. Basic Components of the Model

The multicellular animal organism is a half-chaotic system that develops during the process of ontogenesis. The similarities are large, but the deviations are also important. First of all, the **ontogenesis process itself is not a closed cycle**; i.e., it is not an attractor, let alone a short attractor. The short attractor is for the germline, and this one is still all under selection control. On the other hand, the remaining soma is under the control of selection during part of the ontogenesis process and only until the last multiplication process of the entire system, i.e., the individual (along with the time of caring for their offspring). The influence of selection on the rest of the processes is possible, but it is rather weak and does not necessarily prefer its extension. Soma cells are still half-chaotic systems but also only approximately, because together with the entire organism, they have **two limited sections that differ in the selection control** that affects the entire individual. The selection that directly affects these cells is subject to the control of the entire individual’s interest, but the mechanisms of this control may fail. As observed, the model of the entire individual consists of several interrelated systems that are subjected in various ways to selection control, i.e., maintaining half-chaos. Thus, we will discuss them separately.

### 3.2. Two Parts of an Individual’s Ontogenesis

It may be argued whether an individual’s lifespan is a selection-controlled parameter that is matched to the interests of the species. There are mechanisms that seem to have such a task (e.g., telomeres). Regardless, the lack of clear control of the second part (after the last multiplication) naturally leads to a chaotic explosion, i.e., death.

First of all, ontogenesis does not enter into a closed cycle, i.e., an attractor, and this (according to the experiments of methods 1 and 2, see Section 4.3 and [2]), despite the presence of a large regulation, statistically and inevitably leads to a very long attractor on which the transition to chaos will take place well before its first turn. This reasoning applies to a simplified model with strict determinism, but we know that it is a convenient approximation of stochastic determinism, which will practically prevent entry into the attractor on longer trajectories.

Thus, the mechanism of the half-chaos does not remain, but only the effects of regulation remain: the same effects that were prepared by selection for the first segment of ontogenesis, particularly the one before reproduction into a sufficient number of offspring. (As observed, the border between these segments is blurred.) Again, the wording is too precise, the statistical nature of the process and system subjected to this process softens this conclusion. The in-ice-modularity-based half-chaos type consists of an externally observed attractor of many small attractors in independent “lakes of activity”, i.e., in-ice modules. The support of half-chaos by classic small modules that affect the externally visible attractor in the same manner should be added (see Section 4.4, method 3 in [2]). Processes with a long, uncontrolled attractor do not have to have a strong influence on these modules; they can have a constant direction of influence, slowly shifting the basic parameters of the functioning of other elements. Thus far, this phenomenon has not been studied in terms of half-chaos as the models were too simple for that or these phenomena have not been noticed, but the study of the half-chaos has only just begun. Returning to this hypothetical picture, fluctuations in regulated parameters change circumstances, and over time, more and more regulatory mechanisms will increasingly fall outside the scope of correct operations. Thus, in the second part of ontogenesis, the (internal) mechanisms of the half-chaos maintenance are still present, but there are also mechanisms that spoil their effect, which may even be present to an unnoticeable degree already in the first part. In currently published studies [3], an example of such a mechanism was the growth of larger classic modules in which the transition to chaos was not eliminated by selection that affected the entire organism. These modules, however, grew faster than the network and led to a major crisis.

An individual in the second stage of ontogenesis can also be viewed as a half-chaotic system with an additional slow change (as part of the ontogenesis process) of the parameters on which the regulation depends. Regulation is more and more likely to fail until it causes damages to tear through these protections and explodes into chaos, i.e., death. The sources of the mechanism of this additional slow change may be different: It may be an oversight of the selection, because in the first segment, its influence is small, but it may also be a clock mechanism built in (allowed) by selection that determines the optimal life span of an individual.

### 3.3. The Soma Cell

A multicellular organism arose as a clonal colony of cells that shared tasks among themselves, usually while still sharing the same genome. Kauffman began researching the network by proposing the concept that different tissues are different attractors of the same system [6,7]. This model is still being studied in terms of RBN (e.g., [77,78]) and provides encouraging results. The RBN, however, imposes inconvenient, unnecessary limitations (parameters of edge of chaos) as a result of assumptions that are too strong and not always accurate. It would be good to remove these limitations and apply half-chaos, which allows “chaotic parameters”.

By creating a colony, cells have already had a great deal of mechanisms—an accumulated arsenal of various regulatory and metabolic mechanisms (purposeful information) that undoubtedly form a large, complex network that, as discussed in the previous chapter, is in a half-chaotic state. While the germ-line was under the control of selection throughout the attractor cycle, the rest of the individual, i.e., the soma, has the same time threshold as the entire individual after which the selection has a negligible impact, and its conditions do not necessarily comply with the particular interest of soma cells. Even in the first section of an individual’s ontogenesis, the selection affecting the organism as a whole matches soma (and germ-line) cell changes to the interest of an individual’s survival until it has sufficiently multiplied. This results, in some cases, in even the planned death of certain cells (e.g., apoptosis in the nematode). Obviously, there are regulatory (“police”) mechanisms aimed at ensuring that the functioning and changes within the system’s soma cells are consistent with the selection criterion. Selection then ceases to be merely the maintenance of the half-chaos of the soma cell’s system (i.e., its life and reproductive capacity).

As soma evolve, it begins to play the role of cover of the germline. The germline is a “primitive form” in the model of formation of successive layers of the cover described and illustrated in [44]. This, in principle, is the basic role of soma cells that requires special control of its effectiveness due to the important function of the cover in maintaining the viability of the protected object, which is contrary to the interests of cover elements. This contradiction is particularly strong at the interface between the entire system and the environment, where environmental influences can destroy some elements of the cover, e.g., the epidermis, and the body rebuilds them with the next “kamikazes”. Skin cancer is the most common form of cancer. Soma cells at this interface are most vulnerable to disturbances caused by the external environment, which usually lead to chaos, but they may only destroy “loyalty” mechanisms and leave a half-chaotic state, resulting in cancer.

As with the regulatory mechanisms discussed above, these “police” and “loyalty” mechanisms also have ranges of correct operation that may be exceeded. They can also crash. Deprived of such supervision, soma cells cease to be germline servants; they become independent, and their natural selection returns to the criterion of maintaining their half-chaos and reproductive capacity, but their time horizon for further developments is limited by the inevitable end of their environment—the organism, i.e., death of an individual. The loss of supervision over the most important criterion of functioning—the germline business—is precisely the formation of neoplastic cells. In the second part of ontogenesis, there is no more “germ-line interest”; only “police” and “loyalty” mechanisms operating out of “habit” remain.

Such cells have a similar position to the parasitic cell infecting the organism, with the significant difference that they are particularly difficult to distinguish from the other “polite” cells that make up this organism. Most defense responses against infecting intruders do not recognize the “malfunction” of such a cell and it can reproduce “with impunity”.

### 3.4. Mechanisms of Loss of Control of “Correctness”

The control of the ontogenesis process has two distinct levels. Basic “decisions” are related to the structure and condition of the entire organism. They mark the place and time of events directing the process in the “right” direction, and they define the boundaries of tissues. At this level, there are various regulatory mechanisms that maintain the correct proportions of the body parts. These mechanisms are particularly potent in the development of vertebrates.

The second level contains a cell that, based on a relatively small number of signals from the first level, is able to carry out tasks assigned to it by itself. The first level switches the state of the cell to the appropriate attractor with its signals, and this attractor is already implemented according to the internal scenario in the cell. The cell system contains many possible attractors (for all tissues currently in use in which level one has a key), but it also many attractors to which the entrance is exceptionally opened, unknown or blocked. Among these (normally) inaccessible attractors are atavistic attractors replaced by newer ones, plasticity mechanisms caused by environmental changes, and rescue mechanisms triggered by unique emergency regulations. This is similar to exceptional procedures, e.g., on a ship (and not just a random analogy).

Such exceptional procedures include “genome chaos”. It is possible that entry into this atavistic attractor is blocked, but any blockage can be defeated by increasing largely random damage. Not all such normally unused attractors are led by the path of the “currently applicable procedure”, and entry into such an attractor may be the result of some disturbance and damage caused by this disturbance, which has not been fully regulated by available regulatory mechanisms. Such a form of entry may be also classified as prepared entry caused by stress. The possibility of entering the currently used attractor appears similar, but it occurs in different places or circumstances.

It is hard to expect that the organism has prepared regulatory mechanisms to detect and eliminate such an event for each such exceptional event. The process initiated by such an event follows its own rules, which is damage, usually at the organism level. It encounters many regulatory mechanisms that slow the increase in damage and can even suppress it sufficiently by keeping it low. Often, however, damage increasingly interferes with the remaining, correct processes until it causes a chaotic explosion, i.e., the death of the organism. In the later stage of ontogenesis, this damage develops, and it overcomes the ranges of regulatory mechanisms easier, which, in this section, are no longer controlled (or poorly controlled) by natural selection.

The transition to the “incorrect” attractor does not have to mean that the cell transitions from half-chaos states to death. It still functions, but usually against the interests of the organism, i.e., cell’s environment. In this cell system, the damage was judged to be “small”, and half-chaos remained. If an “incorrect” attractor leads to reproduction, it does so. The process may be interrupted by police mechanisms or insufficient adjustments to the environment—the organism. In the case of where genome chaos arises (as can be on time), a large number of offspring are subject to normal natural selection in the present environment. Most will go proceed from half-chaos to chaos, i.e., die and be eliminated. When an “effective puzzle” of randomly mixed genome happens, the cell lives (retains half-chaos) and continues to reproduce until the organism dies; thus, its environment suddenly changes too radically to continue living. Research (Wright 1999, 2000) shows, however, that this form of genome shuffling is not completely random, but it concerns specific places related to the “failure”, which led to the necessity to switch on the “last rescue” mechanism.

This view is coherent with the “Chaotic Adaptation Theory” (CAT) developed by Tez [1] in 2008 and is expanded in ([12,71,79] and others). Certain titles already show this compatibility: [1]: Cancer is an adaptation mechanism of the aged stem cell against stress; [79]: Complex system perspective in colorectal carcinogenesis; [12]: Cancer is The Chaotic Search For Adaptation To Previously Unknown Environments; and so on. In our paper, we add to this approach a new viewpoint—the half-chaos and deeper understanding of the word chaos in the term genome chaos, as deterministic chaos in the module.

## 4. Model Basics and Half-Chaos Support

This chapter is optional and is intended for those who want to learn more about the model and experiments and the basic results that allowed the detection of half-chaos and the recognition of its basic properties. For many readers, the above-described intuitive picture of half-chaos and its role in the description of a multicellular organism is sufficient, and technical details do not belong to their field. However, the following supplement is also necessary for the cooperation of representatives of biological and medical fields and the simulation of complex systems fields. It explains many of the terms used above, which when accepted on credit only intuitively can turn out to be a bit confusing. Now that you know what these terms are for, it will be easier to know them better.

### 4.1. Description of Networks

Up until this point, a description of a network was not particularly needed, but it will be needed to describe the support for half-chaos in stability creation, which may deform the distribution of damage size. In investigations introducing half-chaos [2], Kauffman networks are used. “Kauffman network” and “Boolean network” were synonymous, but in investigations of Gecow from 2007, more than two equally probable signal variants are additionally used, leaving of the other Boolean network assumptions unchanged. For such slightly modified networks, retaining the name “Kauffman network” was proposed [2,3,22], which makes an important difference between both terms.

**Figure 1:**
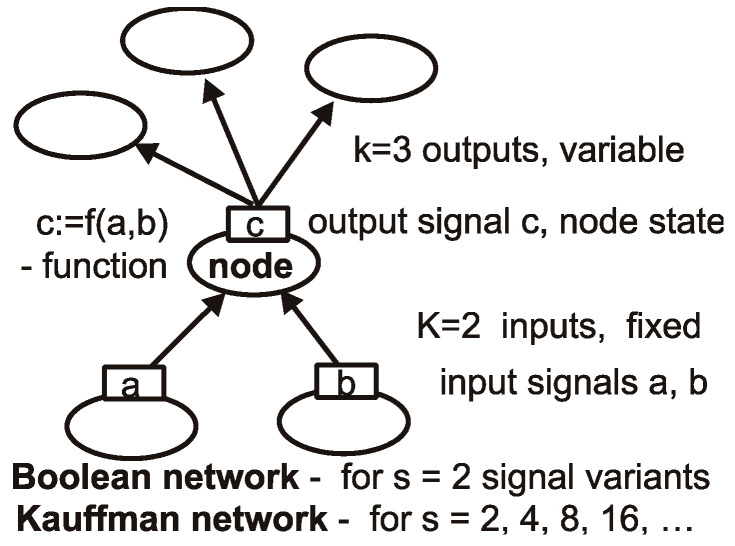
**Network elements: node, its function, signals and links.** Network consists of nodes and links between them. We consider directed networks, and links transmit signals in particular direction. We use s ≥ 2 equally probable signal variants. For s > 2, such networks cease to be Boolean; however, (we propose) they may stay as Kauffman networks. Node has K input links—we keep K fixed for all nodes of a given network (for simplicity of program), but the number of output links k may be different. Node function calculates output signal (node state) using its input signals (input state of node). Here, K = 2 and k = 3, but typically, we use K = 3 and k from 0 to any, but k = 0 normally happens only in er (Erdős-Rényi) network. In sf and ss networks, they are absent. When removing nodes, such nodes with k = 0 may emerge. In the entire autonomous network, there are K × N links.

The Kauffman network is a directed network; this means that each node has **K** inputs and **k** outputs (names K and k are in long use by many researchers). Typically, K is identical for all nodes of the network (for simulation convenience). Input signals are transmitted across input links, and then they are used by node functions as arguments, and the output signal (called node state) is calculated. This state is then transmitted to the next nodes by k output links of our node, which are input links of those next nodes (see Figure 1).

Three **network types** were used: scale-free (**sf**) [5], single scale (**ss**) [80], and classic “random” (**er**) [4].

Typically, a synchronous method of network function calculation is used. In this method, the node’s states from time **t** are taken by links and used as arguments of nodes that receive them for their own functions. In time t + 1, a new state emerges unambiguously in each node of the network as a result of function calculations. All node functions are calculated between t and t + 1. In this method, the sequence of node’s function calculation is not important. Every node in the network has its own state, which might be different for each node. The state of all nodes together is the **state of the network**.

Such a **network is deterministic**; in a twin network (connections of nodes and their functions are all exactly the same), if we begin a second process from the same network state at time t = 0, all consecutive network states will have to be the same as in the first process (the process in our original network). Node functions, the connections of nodes and initial node states are created randomly.

However, to obtain a half-chaotic network, some deviations from such randomness must be performed. To fulfill the “assumption of short attractor”, a complicated algorithm was used, but using this weak assumption, the obtained half-chaos produces an extreme form of the left peak (of damage size distribution), which is not well adequate for a description of nature. An algorithm starting from the “point attractor system” (**PAS** see Section 2.5) is much simpler to implement. It provides half-chaos, leading to a much more adequate form of the left peak. PAS is a stronger assumption than only “short attractor”, however, such form of half-chaos contains mechanism based on “**in-ice-modularity**” (see Section 2.5), that seems to be present in natural systems (see Section 2.5) that seems to be present in natural systems. This type of half-chaos was further investigated in simulation experiments of the evolution of half-chaotic systems. Very similar results were obtained, starting from networks constructed while being based on recognized properties and mechanisms in evolution starting from the PAS.

**Figure 2:**
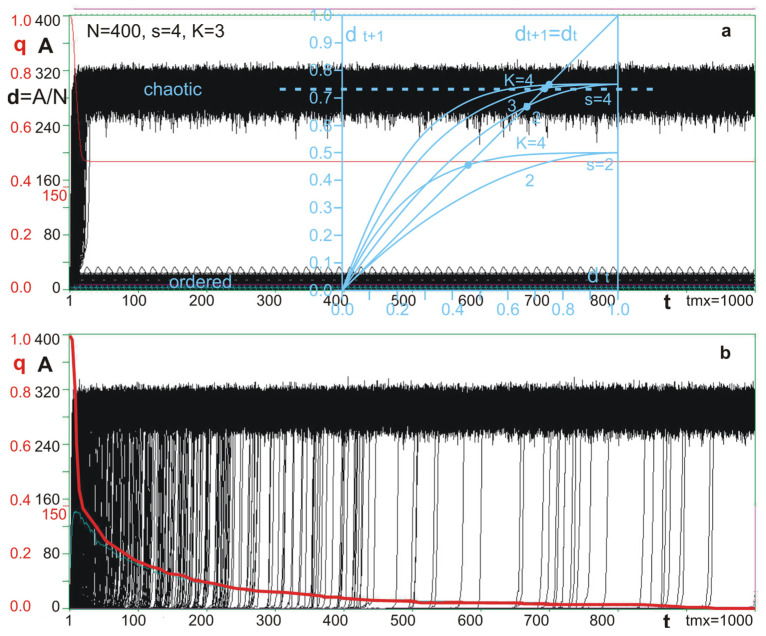
**“Crocodiles” of half-chaotic (a) and chaotic (b) networks. The plot of A(t)**—the number of different node states than in a network without disturbance. Shown plots contain 1200 processes each; it is a complete set of available disturbances of a network of N = 400 nodes and s = 4 when disturbance is a permanent change in function for the initial input state of one node. Red curve (bold for chaotic case b) is a q(t)—part of all processes that stayed still ordered (its damage, d(t), is small, i.e., A(t) < 150; 150 is an arbitrarily taken threshold for a small avalanche). For q and d, it is denoted by the same red scale on the left. As observed, the jump from order to chaos is short, and we call it an “explosion to chaos”. All processes of chaotic network (**b**) exploded before tmx = 1000, but up to tmx, the attractor for the initial network was not found. For a half-chaotic network (**a**), such an attractor was 8, but the teeth in the crocodile’s lower jaw indicate that the attractor of this one particular process is slightly larger but still a short attractor. Explosions into chaos could have happened here until the end of the first attractor cycle. On plot (**a**), a transparent blue “Derrida plot” [9] p. 200, [22] p. 296 is observed. It is d_t+1_(d_t_) calculated from a Derrida-annealed approximation model (constructed for fully random network). The intersection of the curves with the diagonal d_t+1_ = d_t_ is the point of equilibrium called “chaotic Derrida equilibrium”. It is marked for s = 4 and K = 3 by a dashed line, and it also is the maximum of the right peak. For s = 2 and K = 2, there is no such equilibrium; therefore, such parameters lead to an ordered network, and this point is precisely the edge of chaos and order.

In the first series of simulation experiments (described in [2,47]), a simpler case—autonomous networks (no connection to and from environment)—was investigated. A permanent change in one node function was used as the disturbance initializing the damage. The function was changed only for its initial input state (all input signals for this node in time t = 0). The process of functioning with such introduced changes was compared to an undisturbed process and the damage depending on t was observed up until t = **tmx** (maximum time defined arbitrary, typically tmx = 1000). **Damage d**(**t**) = **A** (**t**)/**N** (**N**—number of nodes in the network, typically 400; **A**—number of nodes with different states than in the undisturbed process). Typically, K = 3 and **s** = 4 (number of equally probable signal variants) (Figure 2). These are chaotic parameters; for such parameters, a fully random network is chaotic, but a half-chaotic network has a large left peak and a similarly large right one.

### 4.2. Damage Propagation

A “**coefficient of damage propagation**” (**w**) described in [29,50,51] wider in ([22] Section 2.2.1, [54]), used also, e.g., in [36] eq. 6.2 or [41] eq. 4.8, **has similar meaning to the Lyapunov exponent**. For K = 3 and s = 4, the coefficient w = K(s − 1)/s is equal to 2.25, which produces strong chaos. Phase transitions between order and chaos need w = 1, which is possible only for s = 2 and K = 2—the most extreme case among really complex networks (see [2] Figure 1b). Ordered networks need lower values for s or K and such networks look peculiar.

For s = 4, there are three other than current values of the disturbed function; therefore, for N = 400, there are 1200 different disturbances that may be used. Such complete set of processes depicted in A(t) makes one picture called “crocodile”. For chaotic networks and half-chaotic networks, such pictures (Figure 2) differ significantly. In chaotic networks, “explosions” into chaos occur up to tmx, which means (semi-)all processes will attain a certain damage size near the chaotic equilibrium calculated by Derrida and Pomeau [45] in their annealed approximation model. In half-chaotic networks, the “explosions to chaos” definitely cease to appear after the first rotation of the attractor and a large part of processes (all from the left peak) do not have time to explode. In the main part of tmx length, the picture stabilizes, and P(d) is taken from point tmx. If the damage size is within the range of the left peak, the function’s change remains as an evolutionary change, the network remains half-chaotic and further initiating disturbances are made on the changed network.

When the initializing change is made in PAS, most (over 98%) of the resulting accepted systems (due to small damage) are also PAS. To move the system by evolution to a state other than PAS, attractors smaller than seven were not accepted as evolutionary changes. Nevertheless, the system remained half-chaotic (evolutionary stability of half-chaos) and the attractors did not grow spontaneously. The mechanism of such evolutions is similar to ones described by Kauffman near the edge of chaos—there were small “lakes of activity” in the “ice”, which work as “**in-ice-modules**”. “**Ice**” is a part of network nodes that do not change their states during the process of network calculation (during network functioning). In such small modules (the lakes), the attractors are typically small. In-ice- modularity differ from classic modularity; it is not an effect of the density of connections but of functioning. Typically, there were about three such lakes of activity, and they may freeze for a long period and emerge again in a very similar form.

In a later series of simulation experiments, the networks grew [3]. Additions and removals of nodes involved disturbance changes. Autonomous networks grew from N = 50 to N = 550, but for open networks with **m** = 64 inputs and outputs connecting the network to the environment, N has to be much higher because the gap between peaks of damage size distribution emerges near N = 400 as an effect of the number m of free inputs and outputs [22,30,31,52,56]. Here, networks increased up to N = 4000. Moreover, in growing networks, half-chaos exists, but here, there are many other phenomena that blur the main effect of half-chaos, making the model extremely complicated (the main ones are classic modularity [3]).

### 4.3. Negative Feedbacks

The main source of stability of networks with chaotic parameters is the short attractor that defines the half-chaotic state of networks. However, such stability—resistance on to small random disturbances—is only statistical. Up until the discovery of half-chaos, it was commonly agreed upon that homeostasis is based on negative feedbacks (typical regulation mechanisms) or classic modularity. Such mechanisms and other ones remain as important support of half-chaos in stability, but they lose their independence.

Negative feedbacks are typically taken as the basis of the stability of living entities; even one of the life definitions was based on negative feedbacks [34,35].

**Figure 3:**
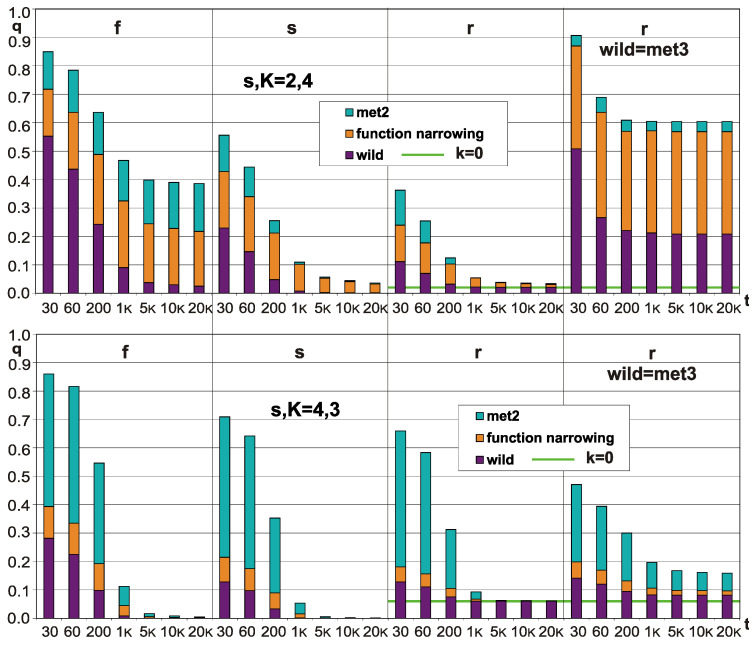
**Effects in q (q—the degree of order) from an increase in negative feedbacks and classic modularity, but without the control of a short attractor** (see met2&3 in [2,47]). The share of negative feedbacks was increased in method 2 by changing some positive feedbacks to negative ones in a fully random network. The side effect of this method was the narrowing of the function, which also had a significant effect on the increase in stability. Both of these factors and the state without these changes are marked. Three types of networks were investigated: sf, ss and er (second letter in diagram). For the er network level of k = 0, it is marked by the green line. In the right column, the results of method 3 are shown, in which modularity was additionally introduced; here, “wild” denotes the effect of introducing modularity itself. N = 400, tmx = 20,000. As observed, a significant increase in stability is observed only for sf 2,4 in met2 and for met3 2,4 even without met2. Parameters s,K = 2,4 and 4,3 result in significant differences in the share of met2 and the narrowing of the function; for 2,4, of the increases in stability is mostly a result of the narrowing of function. Conclusion: of the increase in negative feedback and classic modularity can increase stability in the long term even without the control of a short attractor, but it is an exceptional case. It may be that function narrowing is an important condition in such cases. These tests should be considered as a preliminary diagnosis.

The investigations that result in finding half-chaos start from the thesis that negative feedbacks are enough to make a stable (in statistical sense) network with chaotic parameters. For this question, methods 1 and 2 described in [2,47] were performed but without success. These two methods were based on the conversion of positive feedback to negative ones by a change of functions. The second method was recursive and, therefore, much stronger. In effect, upon short tmx = 60, the stability was radically higher, but when tmx increased to 20,000, nearly all processes exploded into chaos. This depends on parameters s, K and the type of network (Figure 3). Only for scale-free networks, s = 2 and K = 4 it looks like success of the method 2: here, the process of explosion ends, but the narrowing of functions, which is a necessary side effect of the method is the reason of a large part of this effect.

A deep analysis of mechanisms provided two probable reasons: starting from a large fully random network may be an incorrect assumption because in any known, adapted system, evolution starts from a small system and develops into a large system under certain conditions. Shorter attractors reduce probability to an “explosion” into chaos. This remark moves the investigation to method 3, where the thesis that attractors in modules should be short was tested. We will return to this experiment in the next chapter; it, too, was unsatisfactory.

**Figure 4:**
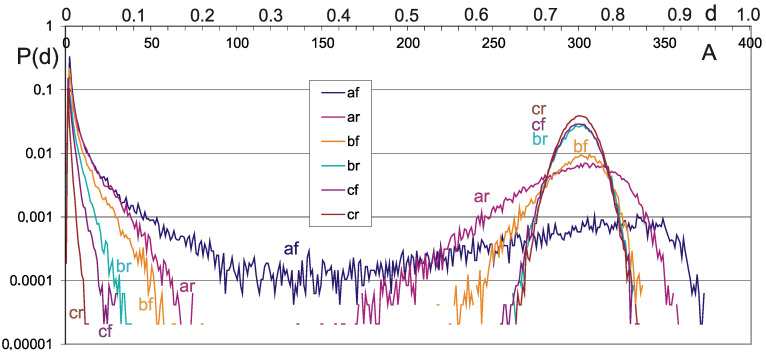
**Two peaks in the damage size distribution of P(d) and the gap between** for network types sf (scale-free) and er (Erdős-Rényi) (second letter of shortcut). Networks parameters: n = 400, s = 4, K = 3, initially PAS. **Influence of regulation by feedbacks**. First letter (a, b and c) indicates the model: A—strong regulation by feedback; b—small regulation by feedback; c—without any regulation (detailed descriptions in [2,47]. The gap and the right peak are blurred only for “af”, i.e., sf network with strong regulation.

The fourth method was to radically remove the attractor problem, but above all, to significantly increase the presence of negative feedback without hindering the interpretation of the presence of function narrowing. The point attractor is extremely short and easy to implement without including any deviation from the randomness of typically controlled parameters. A detailed description of experiments using method 4 can be found in [2,47]. At the first stage of the experiment, the regulation was as great as it was possible for s = 4 and K = 3, and positive results were very strong (Figure 4). Then, minimal regulation was examined, and the positive result was also strong, but it was not as strong as before. At the ending stage of experiment the regulation was absent and the positive result has slightly decreased, but has not disappeared, it remains strong—means it was an effect of point attractor.

The conclusion is that negative feedbacks may significantly support (increase) the stability created by a short attractor, but when alone, they are typically insufficient.

### 4.4. Modularity

The impact of modularity on the stability of a system with chaotic parameters after a small, random disturbance was examined by method 3; however, this is only a very preliminary experiment. It should be further investigated, but it needs new and more advanced models and programs. For such further investigations, the results obtained in method 3’s experiment will be an important premise. Modularity slightly increased stability, but it depends on a few parameters, producing many combinations.

Method 3 (modularity) was combined with method 2 (an increased share of negative feedback and function narrowing). They produced a semi-satisfying effect (Figure 3).

Classic modularity emerges in the investigation of growing autonomous networks. The condition of accumulating a change in the network (addition or removing of a node) is defined for the entire network—damage should be under a certain threshold, i.e., it should be a small change in functioning. However, for a larger network, a large spontaneous module may hide under this threshold. Then, damage inside this module can be chaotic, but such changes can accumulate as evolutionary changes of the entire network. Such modules quickly grow, and a problem emerges when they become greater than the threshold. This is a problem of interpretation: Should such modules be controlled to avoid chaos inside? If yes, then from which point in their growth should they be controlled? This is an important theme for further investigation, as it contains many different aspects leading to opposite results. It is probably unrealistic to provide a general answer to the question if modularity increases or decreases stability; however, modules that are not great produce short attractors, and by this effect, they increase stability. In method 9, in which open networks are examined [3], modularity was the main competitive phenomena of stability created by half-chaos. In this experiment, networks grew up to N = 4000, and the threshold was respectively higher; therefore, modules under the threshold were large. In [81], the role of modularity in evolution was studied in depth.

### 4.5. Function Narrowing

Function narrowing was already known as a feature moving the edge of chaos up in random networks. To measure this feature, Kauffman [9] used P—internal homogeneity for Boolean functions. Later Kauffman et al. [82] used “canalyzing Boolean rules”. For s = 4 in method 2, an additional experiment was performed—iterative function narrowing—which produced a large increase in stability [47] Section 2.5 Figure 18. As was indicated above (and in Figure 3), function narrowing as a side effect of method 2 has significant impacts on the increase in stability. Experiments in method 2 show, however, that function narrowing is not an independent feature; e.g., it also leads to shorter attractors.

## 5. Summary of Model Circumstances of Cancer Cell Formation

Cancer is caused by a disturbance in the function of cells in a multicellular organism, particularly a large disturbance from previously unknown (to cell), environmental change, for which there is no prepared particular reaction for the cell. It leads to stress and applying of “last chance procedure”–genome chaos. This leads to the deactivation of mechanisms that ensure that cell functioning is in line with the interests of the entire organism, and the cell becomes an independent organism that adapts (by natural selection on its level) relative to its own interest. Such views were already proposed by Tez in [1,11,12,71,79]; we continue to propose it here.

Natural selection (on organism level) determines the interest of the entire organism by building guarding mechanisms during evolution. The ontogenesis of multicellular organisms (from the zygote to death) is divided into two sections—until the end of the stage for reproducing a statistically sufficient number of offspring (including care) and the subsequent period. In a first approximation, selection only affects the first segment of ontogenesis, but this statistical boundary is blurred in both directions.

Life is a sustaining the half-chaos [2,3,44], but half-chaos correctly describes only the germline, which is thus controlled by natural selection over the entire closed cycle. The range of this cycle determines the above-mentioned boundary of the control of natural selection in an individual (ontogenesis). The individual is, therefore, not a fully half-chaotic system but a similar one.

Metabolic processes are well described by closed, relatively short attractors. In the first part of ontogenesis, the interest of the germline and the individual is common, and the built-in mechanisms of its control regarding cells remain in the second part of ontogenesis, but the lack of the need to close the cycle allows them to slowly go beyond the scope of correct operations. Regulatory mechanisms struggle with increasing disturbances until it explodes into chaos, i.e., the death of the individual.

Cancer is one of many such processes that break the control of regulatory mechanisms. It concerns a single cell, which as a result of a disturbance, however, has not lost its ability to reproduce and has ceased to act in accordance with the interests of the whole, becoming an independent half-chaotic system limited only by the need to maintain a half-chaotic state and the reproduction mechanism. In this aspect, it is no different from a parasite that infects the body, but the body has more difficulty in recognizing it as a parasite. Such a cell, engaging (in a less controlled manner) various rescue mechanisms, proceeds into normally unused attractors.

Soma, which is a form of germ-line cover [44], is by nature more vulnerable to destructive environmental effects, making cancer appear more frequently here.

Currently, simple artificial networks are used to model these phenomena, mainly Kauffman networks, but they do not have the possibility to build their structure as a result of their own functioning. Even though the models under consideration are already very complicated, those networks have limitations. They provide basic, general properties of complex networks, their functioning and evolution, but the self-creation properties of their structure along with eliminating the qualitative difference between functioning nodes and signals and links are missing in those networks. Those properties are necessary to achieve a satisfactory similarity of the model networks and the modeled phenomena from real biological systems.

The word chaos in the term genome chaos has different meanings than in deterministic chaos. We have analyzed these differences based on experiments described in [3], which led to a rapprochement: the term chaos in genome chaos can be treated as deterministic chaos in the module of the system that models the cell.

## Data Availability

The data is included in the paper.
